# Assessment of time irreversibility in a time series using visibility graphs

**DOI:** 10.3389/fnetp.2022.877474

**Published:** 2022-10-04

**Authors:** Małgorzata Andrzejewska, Jan J. Żebrowski, Karolina Rams, Mateusz Ozimek, Rafał Baranowski

**Affiliations:** ^1^ Cardiovascular Physics Group, Physics of Complex Systems Division, Faculty of Physics, Warsaw University of Technology, Warszawa, Poland; ^2^ Institute of Cardiology, Warszawa, Poland

**Keywords:** irreversibility, visibility graphs, heart rate variability, repolarization, kld

## Abstract

In this paper, we studied the time-domain irreversibility of time series, which is a fundamental property of systems in a nonequilibrium state. We analyzed a subgroup of the databases provided by University of Rochester, namely from the THEW Project. Our data consists of LQTS (Long QT Syndrome) patients and healthy persons. LQTS may be associated with an increased risk of sudden cardiac death (SCD), which is still a big clinical problem. ECG-based artificial intelligence methods can identify sudden cardiac death with a high accuracy. It follows that heart rate variability contains information about the possibility of SCD, which may be extracted, provided that appropriate methods are developed for this purpose. Our aim was to assess the complexity of both groups using visibility graph (VG) methods. Multivariate analysis of connection patterns of graphs built from time series was performed using multiplex visibility graph methods. For univariate time series, time irreversibility of the ECG interval QT of patients with LQTS was lower than for the healthy. However, we did not observe statistically significant difference in the comparison of RR intervals time series of the two groups studied. The connection patterns retrieved from multiplex VGs have more similarity with each other in the case of LQTS patients. This observation may be used to develop better methods for SCD risk stratification.

## 1 Introduction

Physiological systems, such as the human body, for example, are considered complex (Seely and Macklem, 2012). Such systems use energy to build increasingly complex and ordered structures. This ability of self-organization is related to the directivity of energy flow and the irreversibility of the processes taking place (Costa et al., 2005). Healthy organisms are believed to work under conditions that are far from equilibrium. Such states are characterized by the production of entropy. This results from the fact that organisms form an ordered structure during development, therefore, for the second law of thermodynamics to be preserved, this process must be balanced by the production of entropy (Seely and Macklem, 2012). There is also a hypothesis that the assessment of this production can be used to diagnose the state of dynamic equilibrium of the organism (Seely and Macklem, 2012).

In physiology, the condition in which the stable conditions of the internal environment of the body are maintained is called homeostasis. To survive, the organism requires the maintenance of an appropriate concentration of many quantities, such as nutrients, oxygen concentration and various ions. In addition, the maintaining of appropriate temperature and blood pressure levels is required (Chladekova et al., 2012). There are gradients of these quantities in the body, which are related to metabolism and have a significant impact on the rate of production of entropy of the system. In turn, it is known from statistical physics that this rate is related to the irreversibility of the studied processes (Seely and Macklem, 2012).

In the case of healthy and young organisms are characterized by a greater complexity, related to the greater adaptability of such organisms (Seely and Macklem, 2012). The decrease in the possibility of self-organization, and, therefore, the decrease in irreversibility over time, can be associated with aging of the organism or may be due to diseases (Costa et al., 2005). Many studies show that such a decrease may be associated with a decreased heart rate variability (Seely and Macklem, 2012). In statistical terms, a signal can be considered irreversible when its statistical properties change after reversing the passage of time.

Different studies, e.g. (Jose and Taylor, 1969), have showed that pharmacological blockade of cardiac autonomic control reduces heart rate variability and increases its basal beating rate in humans. This is due to autonomic control by both sympathetic and parasympathetic nervous system and dominant inhibition of cardiac pacemaker by the vagus nerve in healthy humans. This natural rate of an unperturbed sinus node is called intrinsic heart rate (IHR) and it declines with age (Jose and Taylor, 1969; Opthof, 2000). Assessing IHR may provide an insight into the pathological mechanisms and help with antiarrhythmic therapies (Marcus et al., 1990). The difference between IHR and mean HR defines an operational range for neural and hormonal regulation. As this difference diminishes in time, it is observed that heart rate variability measures will also decline with age (Jandackova et al., 2016).

Regarding the Long QT syndrome (LQTS), an inherited proarrhythmic cardiac abnormality, the clinical target of our research is not to simply diagnose the disease. There are simple ECG-based methods to distinguish patient from healthy individuals (Schwartz et al., 2012). We rather refer to the fact that the patients with LQTS are more susceptible to develop fatal cardiac arrhythmias (Mathias et al., 2013). It follows that LQTS is a risk factor for sudden cardiac arrest (SCA) (Goldenberg et al., 2011). The clinical goal of this study is to assess irreversibility in a group that has an arrhythmic substrate (Vijayakumar et al., 2014). This substrate is present in all LQTS patients, but its severity is unknown. Risk stratification in this group will be a difficult task which requires a prospective study.

On the other hand (Suboh et al., 2019), have shown that the use of artificial intelligence (AI) algorithms can predict sudden cardiac arrest from ECGs with up to 92% efficiency. This means that the even a short ECG strip, and most notably the normal sinus rhythm variability contains information about the possibility of SCA. AI is usually non-conclusive, and it is difficult to relate the results of its performance to the measured parameters. Explainable artificial intelligence is evolving (Samek et al., 2019), but the information it provides is formulated in the feature space of the model, e.g., the convolutional network, and not in the concept space of traditional ECG or HRV analysis. However, the exceptionally high success rate of the AI methods convinces that there is reason to study individuals at increased risk of sudden cardiac arrest and compare them with healthy individuals. The key feature of the normal cardiac rhythm, which is believed to carry important clinical information is its complexity. The concept of complexity is complicated and can be explained using different methods, e.g., fractal analysis, entropy, or irreversibility (Fiskum et al., 2018). When studying heart rate variability, the question of which concept space will be the best to describe the patient’s clinical condition recurs. Measures of irreversibility applied here can be used to differentiate groups, and they become interesting candidates to better assess the risk of SCA and improve patient management, to increase life expectations and reduce mortality.

In this paper, we analyze only a part of the physiological network of the human, namely, we assess time irreversibility of time series taken from ECG recordings. The purpose of this paper is to analyze irreversibility in a group of patients with the LQTS (Long QT syndrome) and compare them to a group of healthy persons to identify dynamical correlates of the arrhythmogenic substrate. However, comparing time irreversibility descriptors presented below with standard statistics shows that both approaches provide similar results in distinguishing between groups (Figure 6 below). The mean and standard deviation of QT intervals are greater in the LQTS group. These indicators are simplified, however, and the use of irreversibility over time provides a way to distinguish between differences in the dynamics caused by reversible and irreversible processes (Lacasa et al., 2012). The presence of time irreversibility indicates the existence of nonlinear processes such as dissipative chaos (Li et al., 2021). Ilya Prigogine discovered the existence of dissipative structures (Prigogine, 1978), which are spontaneously self-organizing complex system states that arise far from equilibrium. Living organisms, including humans, can be looked at as dissipative structures far from a thermodynamic equilibrium (Li et al., 2021). They are characterized by a high degree of complexity, which can be estimated using non-linear properties of human heartbeat (Seely and Macklem, 2012). To compare the results for time asymmetric patterns with irreversibility measures using KLD, we calculated the Porta and Guzik indices.

LQTS is a genetically determined dysfunction of ion channels or the proteins that regulate them. This disease leads to serious symptoms, including fainting or loss of consciousness. It can also cause sudden cardiac arrest (SCA). A prolonged QT interval can be acquired or congenital. The clinical course of the disease varies depending on which gene has been mutated. The most common types of LQTS are LQTS 1, 2 and 3. In the case of LQTS type 1, which is most of the cases we study in this paper, the mutation disrupts the slow potassium current (Seebohm et al., 2008). Symptoms of the disease most often occur during exercise, in contrast to, for example, LQTS2, where they are induced during increased catecholamine release in early morning (Wilde et al., 1999).

Complex networks are increasingly used in various fields of science. Currently, graphs are used in many practical problems, including in computer networks, where the representation of the network in the form of a graph facilitates the routing of data packets on the Internet (Oehlers and Fabian, 2021), in medicine to study the spread of viruses (Keeling and Eames, 2005; Alarcón-Ramos et al., 2018), or research on the dynamics of social networks, e.g., the spread of rumors (Agliari et al., 2017). Historically, Kullback-Leibler divergence (KLD) was proposed for measuring time asymmetry in the beginning of the 1950s and during the next decade its relation to entropy production was shown (Gaspard, 2004; Parrondo et al., 2009). In our study, we used visibility graphs (VG) methods (Lacasa et al., 2008) to assess the time irreversibility of selected time series. VG allow to map time series to the form of graphs. This makes it possible to study the information contained in such records with the use of complex network research tools. In this way, graph theory can be used to study nonlinear signals (Lacasa et al., 2008).

This paper is constructed as follows: in Section 2, we introduce time irreversibility methods both for one dimensional time series and multivariate time series. In Section 3, we describe the data, which were used for our analysis, and introduce our methodology for preparing data extracted from ECG recordings from the THEW database (University of Rochester Medical Center, 2022; University of Rochester Medical Center Healthy Individuals, 2022). In Section 4, we present our results for nighttime recordings of healthy people and of patients with LQTS. In Section 5, we conclude.

## 2 Methods

### 2.1 Visibility graphs

Graphs were also looked at from the medical point of view (Mason and Verwoerd, 2007). The authors of the publication (Lacasa et al., 2012) showed that the increase in entropy per unit time can be described by determining the Kullback-Leibler divergence (KLD), usually denoted KLD (*p* || *q*) for a given random variable x and probability distributions p(x) and q(x) . However, this measure gives only the lower bounds of entropy production (Lacasa et al., 2012). For two probability distributions p and q , describing the process in accordance with and contrary to the passage of time, KLD it is given by the relationship (Lin, 1991):

$$KLD\left(p\|q\right)=\sum_{x}p\left(x\right)\log\frac{p\left(x\right)}{q\left(x\right)}.$$ (1)

**FIGURE 1:**
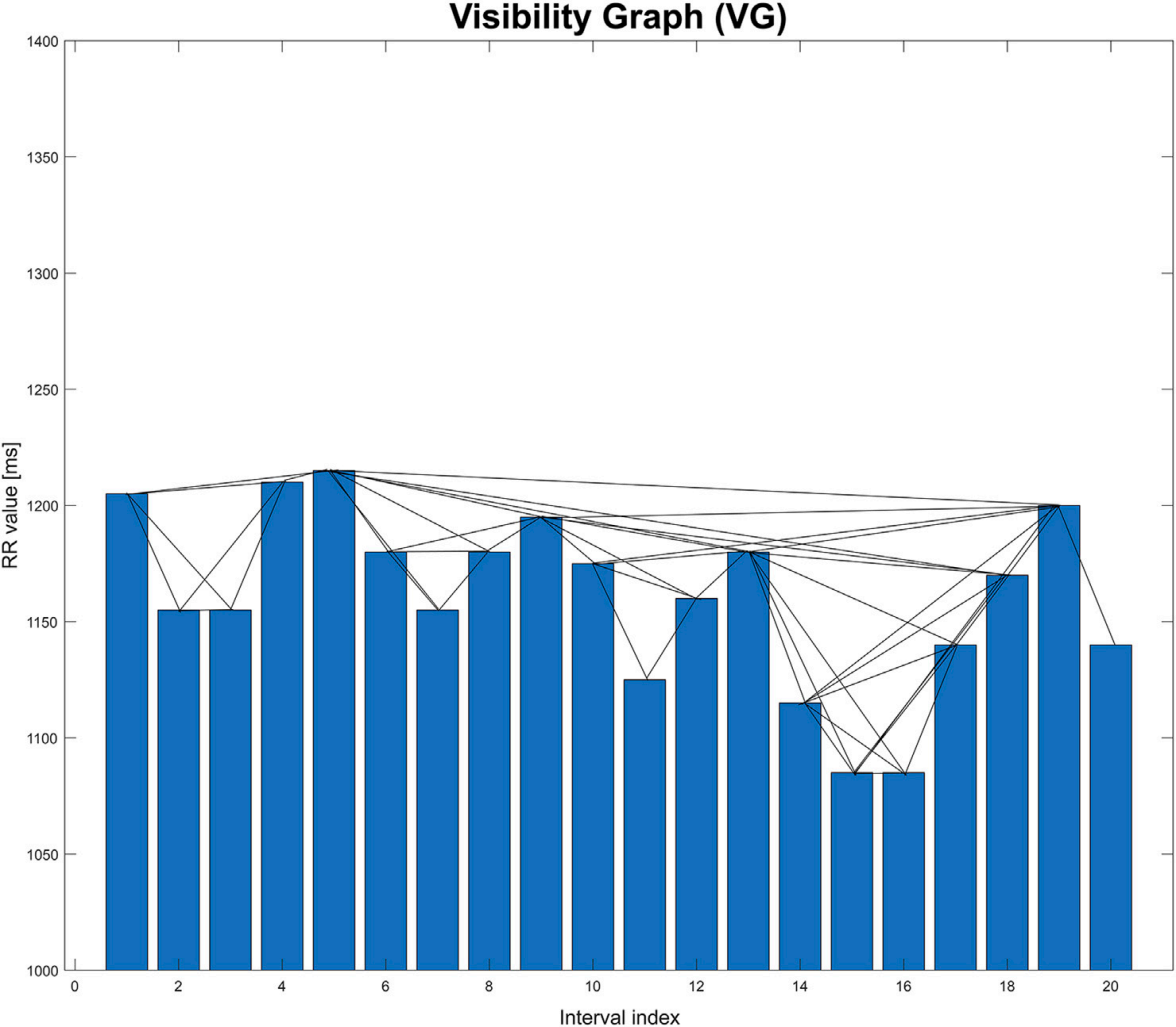
Graphical illustration of visibility graph (VG). This graph is based on an extract from one of the records studied in the paper.

Such a graph is created by connecting the vertices that meet a specific visibility criterion. Figure 1 shows an example of a time series in the form of a bar graph. When analyzing human heart rhythm records, each bar corresponds to a single value of the RR interval (measured as the time between two successive R-waves in the ECG trace). Each such interval is also the vertex of the graph, into which the time series is transformed (Iacovacci and Lacasa, 2016).

Two vertices are connected to each other when the heights of the corresponding bars meet the following visibility criterion (Lacasa and Flanagan, 2015). For the time series $S={\left\{x\left(t\right)\right\}}_{t=1}^{T}$ for each element $x_{i}\left(t\right)$ being the vertex of such a graph, two vertices i and j are connected by an edge, if each different $x_{k}\left(t\right)$ satisfies condition:

$$x_{k}<x_{i}+\frac{k-i}{j-i}\left[x_{j}-x_{i}\right],for\;each\;i<k<j.$$ (2)

### 2.2 Horizontal visibility graphs

**FIGURE 2:**
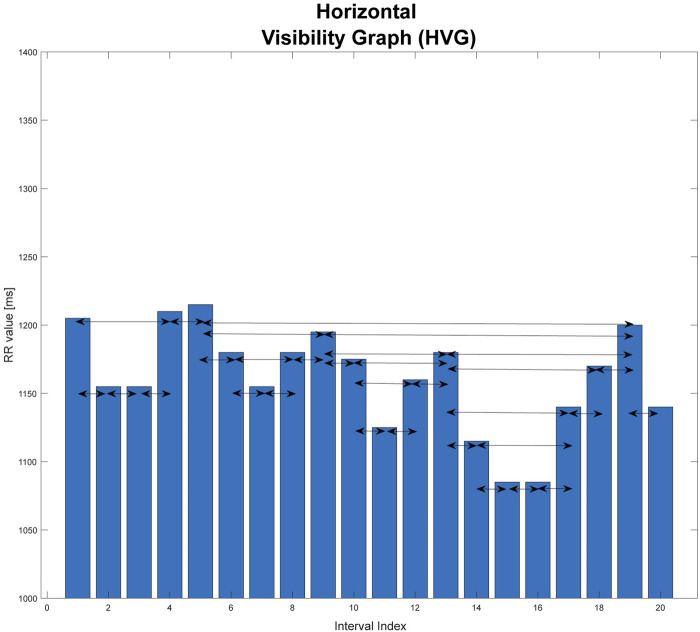
Graphical illustration of horizontal visibility graph (HVG). This graph is based on an extract from one of the records studied in the paper.

Another type of graph is the horizontal visibility graph. It differs from the basic version in that, in this case, two vertices are connected to each other only if they can be joined together in a bar graph of the time series by a horizontal line without intersecting the vertices between them (Lacasa et al., 2012). An example is shown in Figure 2.

In general, for the time series $S={\left\{x\left(t\right)\right\}}_{t=1}^{T}$ the following condition of horizontal visibility can be written (Lacasa and Flanagan, 2015):

Two vertices $x_{i}\left(t\right)$ of the graph are connected with each other if and only if the following relation is satisfied:

$$x_{i},x_{j}>x_{n},\;for\;every\;i<n<j.$$ (3)

### 2.3 Directed horizontal visibility graphs

This is a graph that is an extension of the horizontal visibility graph. The direction of the flow of time is taken into consideration. The temporal arrow is considered by using directed graphs. For each vertex, you can specify the edges that enter it from the vertices that precede it, and the edges that connect it to the next vertices that follow it in time. The direction of connections is consistent with the passage of time (Lacasa and Flanagan, 2015).

The degree of the vertex k(t) consists of the following sum:

$$k\left(t\right)=k_{in}\left(t\right)+k_{out}\left(t\right).$$ (4)

**FIGURE 3:**
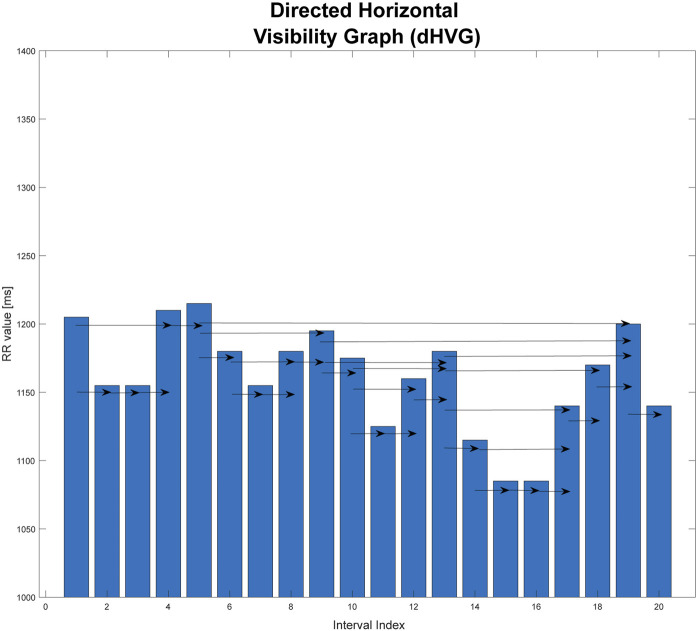
Graphical illustration of directed horizontal visibility graph (dHVG). This graph is based on an extract from one of the records studied in the paper.

$k_{in}\left(t\right)$ is the number of edges entering a given vertex, associated with vertices in the past. On the other hand, $k_{out}\left(t\right)$ is defined as the number of edges emerging from a given vertex. This is related to the connections of a given vertex with the “future” elements of the time series (Lacasa and Flanagan, 2015). An example of such a graph is shown in Figure 3.

The analysis of the dHVG allows the use of information on the degree distributions of the incoming and outgoing vertices. Based on the difference in these distributions, the degree of irreversibility of the time series tested can be estimated. This difference can be interpreted as the distance (in the sense of distributions) between the probability distributions of the input vertices $P_{in}\left(k\right)$ and that of the output $P_{out}\left(k\right)$ . Generally, *P*(*k*) is the fraction of all nodes in the network that have degree k and it describes the probability that a randomly selected node will have degree k (Lacasa and Flanagan, 2015).

One of the measures that allows to describe the difference between the distributions $P_{in}\left(k\right)$ and $P_{out}\left(k\right)$ is the Kullback-Leibler divergence:

$$KLD\left[P_{out}\left(k\right)\|P_{in}\left(k\right)\right]=\underset{k}{\sum }P_{out}\left(k\right)\cdot \ln\frac{P_{out}\left(k\right)}{P_{in}\left(k\right)},$$ (5)

where:

k - vertex degree and $k=k_{in}+k_{out}$

*P*_*in*_(*k*)- degree distribution of input vertices

$P_{out}\left(k\right)$ - degree distribution of exit vertices.

In statistical physics, the measure KLD can be used to measure the time irreversibility of non-equilibrium processes and to estimate the entropy production during such processes (Lacasa et al., 2012). It was shown in (Lacasa et al., 2012) that this measure enables to distinguish discrete time series obtained from reversible and irreversible time series.

The signal is invertible when:

$$\lim_{n\to \infty }KLD\left[P_{out}\left(k\right)\|P_{in}\left(k\right)\right]=0.$$ (6)

where n is number of vertices in the graph. In this case, the probability distributions $P_{in}\left(k\right)$ and $P_{out}\left(k\right)$ are equal.

For stationary signals, KLD is the lower limit of the non-equilibrium entropy production during the time evolution of the process.

The Jensen-Shannon Divergence (JSD) is a measure of divergence based on KLD. Its main advantage is that, in contrast to the Kullback-Leibler divergence, it always has a finite value, which allows to avoid infinity obtained when calculating KLD (Nielsen, 2020).

JSD can be determined as the mean KLD divergence of the distributions $P_{in}\left(k\right)$ , $P_{out}\left(k\right)$ and their mixed distribution $M=\frac{P_{in}\left(k\right)+P_{out}\left(k\right)\;}{2}$ (Nielsen, 2020):

$$JSD\left[P_{out}\left(k\right)\|P_{in}\left(k\right)\right]=\frac{1}{2}(KLD\left[P_{out}\left(k\right)\|M\right]+KLD\left[P_{in}\left(k\right)\|M\right].$$ (7)

After the substitution, the final formula is:

$$\begin{array}{l} JSD\left[P_{out}\left(k\right)\|P_{in}\left(k\right)\right]=\\ =\frac{1}{2}\left[\underset{k}{\sum }P_{out}\left(k\right)\cdot \ln\frac{P_{out}\left(k\right)}{\frac{1}{2}\cdot \left[P_{out}\left(k\right)+P_{in}\left(k\right)\right]}+\underset{k}{\sum }P_{in}\left(k\right)\cdot \ln\frac{P_{in}\left(k\right)}{\frac{1}{2}\cdot \left[P_{out}\left(k\right)+P_{in}\left(k\right)\right]}\right]. \end{array}$$ (8)

### 2.4 Multivariate methods

Now, we consider an M-dimensional real valued time series. Using such data, an M-layer Multiplex network is constructed (Lacasa et al., 2015). In our case, we have a set of α data ( α=3 for the intervals RR, QT and DI of the ECG trace). Each of them is a series of real data from index 1 to the length of signal N . For each of them, we construct the HVG in accordance with the single-layer algorithm (Lacasa et al., 2012; Lacasa et al., 2015; Lacasa et al., 2017). The Multiplex Visibility graph is created in such a way that it is described by a matrix $A=\left\{A^{\left[1\right]},\;A^{\left[2\right]},\;A^{\left[3\right]}\right\}$ , the elements of which are the adjacency matrices of the VG of each of the examined data sets (in our case, the intervals RR, QT and DI).

Average edge overlap <o> is defined as follows (Lacasa et al., 2015):

$$<o>=\frac{1}{K}\sum_{i,j}o_{ij},o_{ij}=\frac{1}{M}\sum_{\alpha }a_{ij}^{\left[\alpha \right]}.$$ (9)

where K is the total number of edges and $o_{ij}$ is the overlap of the edges between the vertices i and j situated in different layers. It is defined as follows: we sum for each pair ij the appropriate terms in the adjacency matrix (equal to 1 if these vertices are connected to each other in each layer, 0 otherwise). This is then normalized by the number of layers. $o_{ij}=0$ , when the nodes i and j are not connected to each other in any layer, and 1 when they are in all of them. Next, we sum these values over i, j and average over the number of i, j pairs. Thus, the more similar the connection patterns in the layers are, the larger <o> we obtain. <o> equals 1 , when all the layers are identical (Lacasa et al., 2015; Lacasa et al., 2017).

We compute the adjacency matrices for directed graphs according to the passage of time and after inverting the sequence of the records. Then, we will obtain matrices which in KLD are used to determine $P_{in}\left(k\right)$ —the degree distribution of input vertices and $P_{out}\left(k\right)$ —the degree distribution of output vertices. Having these two data sets, for each of them we calculate the average edge overlap and then calculate the absolute value from the difference of these values. Directed average edge overlap:

$$davo=abs\left(<o_{in}>-<o_{out}>\right).$$ (10)

We also used interlayer mutual information (IMI) (Lacasa et al., 2015) as another measure of quantification of the presence of interlayer correlations. For two layers α and β, IMI between the degree distributions $k_{\alpha }$ and $k_{\beta }$ is defined as:

$$I_{\alpha ,\beta }=\sum_{k_{\alpha }}\sum_{k_{\beta }}P\left(k_{\alpha },k_{\beta }\right)\log\frac{P\left(k_{\alpha },k_{\beta }\right)}{P\left(k_{\alpha }\right)P\left(k_{\beta }\right)}$$ (11)

During the calculation of IMI, after the division of the signal into non-overlapping windows of 600 interval length, we used the EMD method (Stallone et al., 2020) to remove the trend from the data. To do so, we separated the last four IMFs and their sum we subtracted from the signal. This was required for proper calculations of mutual information (Hoyer et al., 2005).

### 2.5 Asymmetry indices

Porta’s Index (*P*%) (Porta et al., 2008) compares the number of negative increments between consecutive members of the time series with the number of all non-zero increments. It is defined by the formula:

$$P\%=\frac{N\left(\Delta RR^{-}\right)}{N\left(\Delta RR\neq 0\right)}*100\%.$$ (12)

This index can range from 0 to 100%. The irreversibility over time is implied by *P*% values significantly different from 50%. Moreover, *P*% values greater than 50% indicate that the number of negative increments ΔRR^-^ in the signal $RR_{i}-RR_{i+1}$ is greater than the number of positive increments ΔRR^+^.

To make the values of this index more readable, below we subtract 50 from all values obtained for the different cases studied. In this way, the zero of this index indicates a completely reversible time series. We treat the next index (described below) in the same way.

Guzik’s index (G%) (Piskorski and Guzik, 2007; Porta et al., 2008) is determined as the ratio of the sum of the squares of the positive differences $RR_{i}-RR_{i+1}>0$ to the sum of all differences $RR_{i}-RR_{i+1}$ in the signal squared. This index can also be defined as the ratio of the sum of squared of positive differences $RR_{i}-RR_{i+1}>0$ from diagonal in the Poincaré plot (this is a scatter plot describing the dependence $RR_{i+1}=f\left(RR_{i}\right)$ (Piskorski and Guzik, 2007)) signal to the distance of all ΔRR from the diagonals. It is given by the formula:

$$G\%=\frac{\sum_{i=1}^{N\left(\Delta RR^{+}\right)}\Delta RR^{{+}^{2}}\left(i\right)}{\sum_{i=1}^{N\left(\Delta RR\right)}\Delta RR^{2}\left(i\right)}*100\%.$$ (13)

G% can take values from 0 to 100%. The signal irreversibility over time is implied, as in the case of the Porta’s index, by G% values significantly different from 50%. For clarity, we subtract 50 form the value obtained for each case studied. This is the same procedure that we used for Porta’s index.

## 3 Data and methodology

Two databases from the THEW Project (University of Rochester Medical Center, 2022; University of Rochester Medical Center Healthy Individuals, 2022) were used to provide the RR, QT, and the DI intervals (diastolic interval - the time between the end of the T segment and the beginning of the next QRS complex). We used the following THEW databases: E-HOL-03-0202-003 (202 ECGs of healthy individuals) and E-HOL-03-0480-013 (480 ECGs of the Long QT Syndrome patients forming 4 subgroups by genotype).

In this paper, we analyze a subgroup for each of these databases: It consists of 61 (38 women) LQTS patients and 114 (59 women) healthy persons. The range of age is limited to 18–60 years.

To calculate the RR and QT intervals, firstly, the R waves in the ECG signals had to be obtained. To achieve that, proper annotation files derived from the THEW database were placed onto the signals and then filtered to delete those R waves that had not been annotated as either normal or arrhythmic. This method, however, resulted in the R waves being misplaced by an irregular offset, rendering them incorrect. To compensate for these offsets a hybrid algorithm was developed. The algorithm used the R waves detection toolset available in the Neurokit2 package for Python 3, which allowed for correct detection of the R waves. However, the results of this operation would have had to be manually selected for each separate file, as this method gave no information if the peak was normal, arrhythmic or of other kind. In addition, it was oversensitive towards labelling other types of waves as R waves when the signal was of especially bad quality. The hybrid algorithm combined the two methods, i.e., using the annotation files and the Neurokit2 toolset, and compared the results of both, deleting the offsets from the first method. Based on the obtained R waves, the wave detection toolset available in the Neurokit2 package was used to find other waves, among which there were the Q waves and T waves offsets. To obtain the RR and QT intervals, the difference between R(i) and R(i+1) as well as the differences between Q(i) and T_offset(i) were calculated.

KLD values and multivariate methods were determined using Matlab R2021b, while statistical tests and graphs were done using OriginPro 2021b.

All signals were divided into non-overlapping windows of length 600 intervals. The numbers next to the pairs of boxplots on Figures 6–11 are the corresponding *p*-values (Kolgomorov-Smirnov test). This non-parametric test was chosen because the data distributions do not meet the criterion of fitting a normal distribution (Shapiro-Wilk normality test).

KLD was calculated only for the nighttime recordings of the heart rhythm. Because of the different time for every patient for going to sleep, for each case the period of observation was selected using the average RR value over time (Gierałtowski et al., 2012; Żebrowski et al., 2015). These records were also analyzed with the use of windows (the tested signal was divided into adjacent, non-overlapping windows). Windows with the lengths of 400, 600, 900, 1,200 and 2000 intervals were used. A window with the length of 600 RR intervals was finally used for the analysis, this value was considered optimal. The selection of such a window width was made after analyzing the results for other window widths. For the 600 interval window length, we obtained the best results in comparing the study groups. The window with a width of 400 intervals is too short for the method to give perfect identification of irreversibility (Zanin and Papo, 2021), while the results for windows of 900, 1,200 and 1800 intervals showed a dispersion of the results which was too large. The result is the average obtained from all windows of a given length into which the time series was divided into.

**FIGURE 4:**
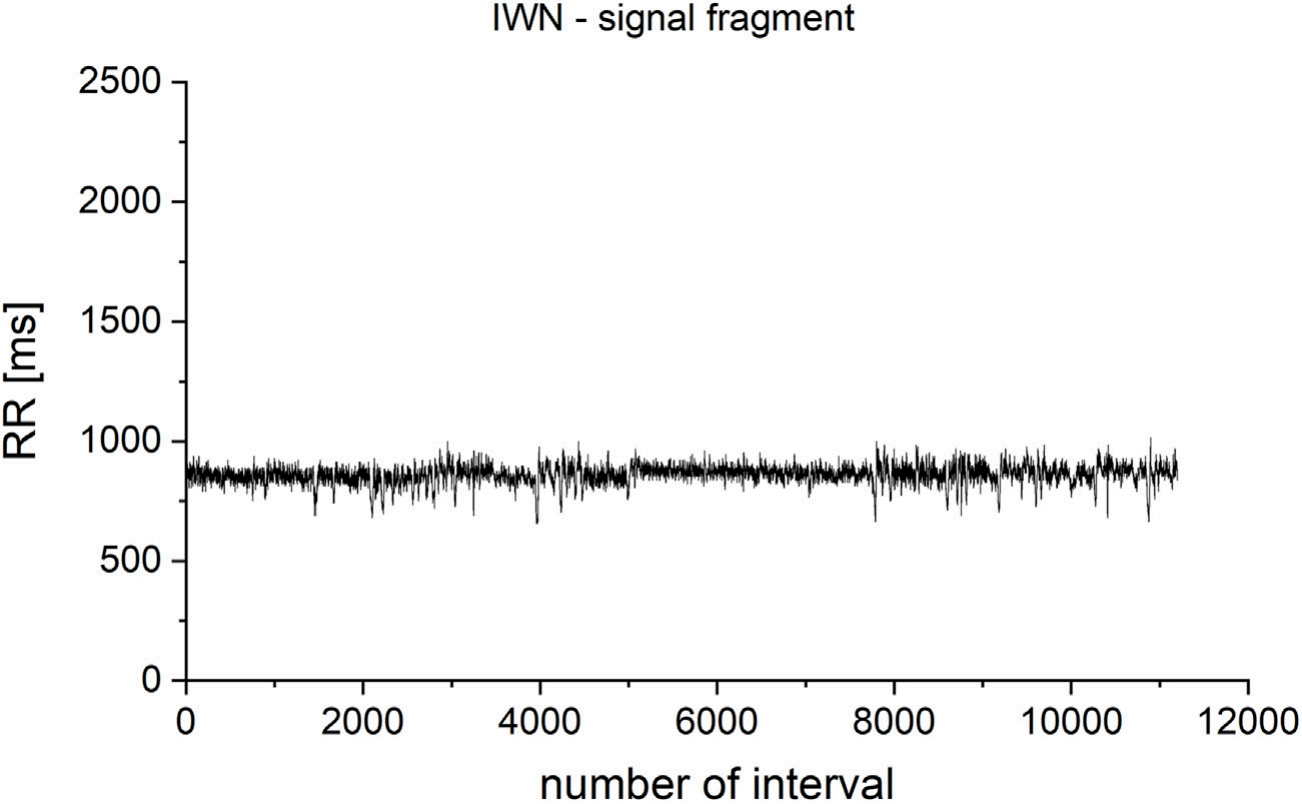
Fragment selected from the IWN patient’s heart rhythm record. The total analyzed record length was from *N* = 100 to *N* = 11,200 iterations. Successive fragments of increasing length were selected, starting at the beginning of the time series.

The dependence of the results of irreversibility measures on the length of the analyzed time series was also checked. For this purpose, a fragment of a record of the heart rhythm of a healthy male from the database of the Institute of Cardiology (patient IWN, Figure 4) was checked. The total record length analyzed in the study was from *N* = 100 to *N* = 11,200 intervals. Successive fragments of increasing length were selected, starting at the beginning of the time series.

**FIGURE 5:**
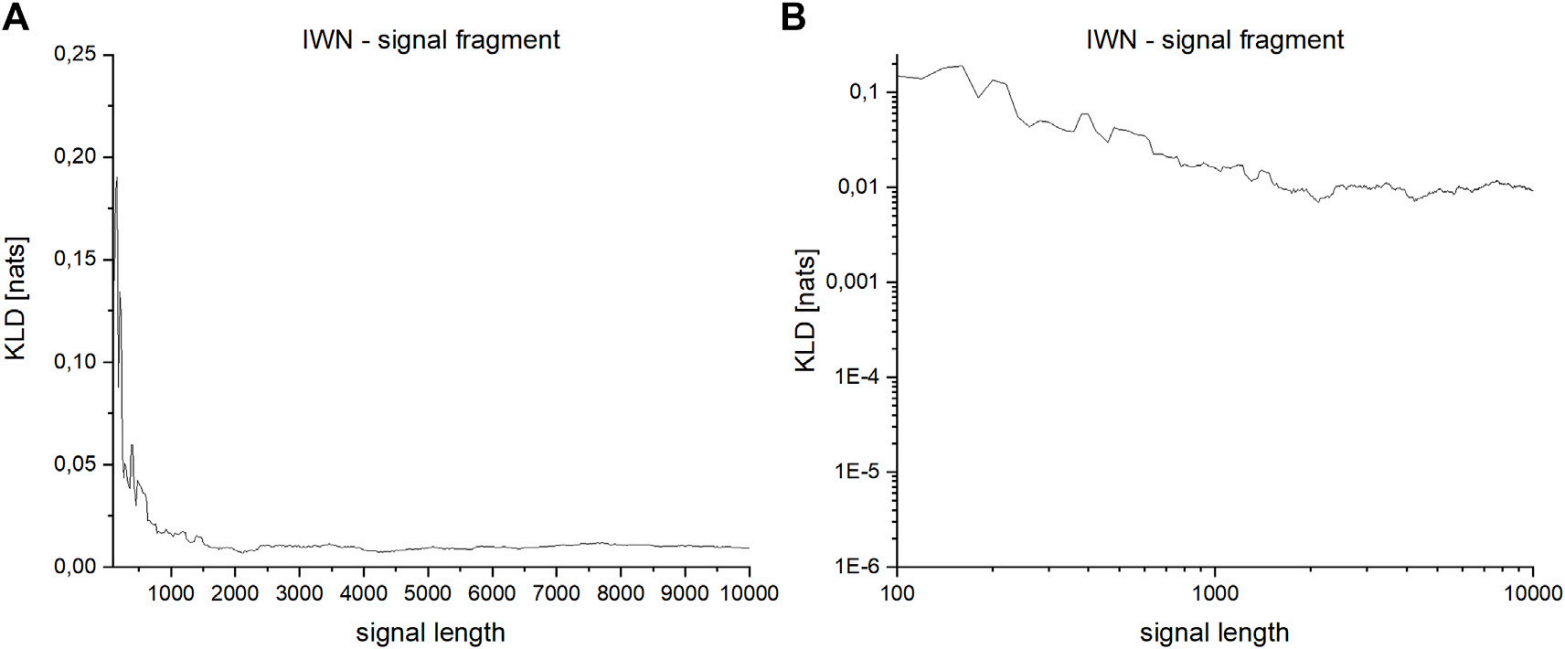
**(A,B)**—Dependence of KLD on the length of the series for a fragment of the signal derived from the heart rhythm of a healthy person: **(A)**—linear scale, **(B)**—logarithmic scale.

For windows of the length 600 intervals, KLD still do not fully stabilize, but it is the optimum between the correctness of the method (low dependence on the length of the tested time series), the quality of the obtained results (statistical significance measured in the Kolgomorov-Smirnov test using the *p*-value value), and the time required to carry out the calculations (Figure 5A). To represent the dynamics more clearly for shorter signals, for which there is a large difference between the analyzed values, the results are also presented in a logarithmic scale (Figure 5B).

## 4 Results

### 4.1 Assessing time irreversibility of nighttime recordings using VG

**FIGURE 6:**
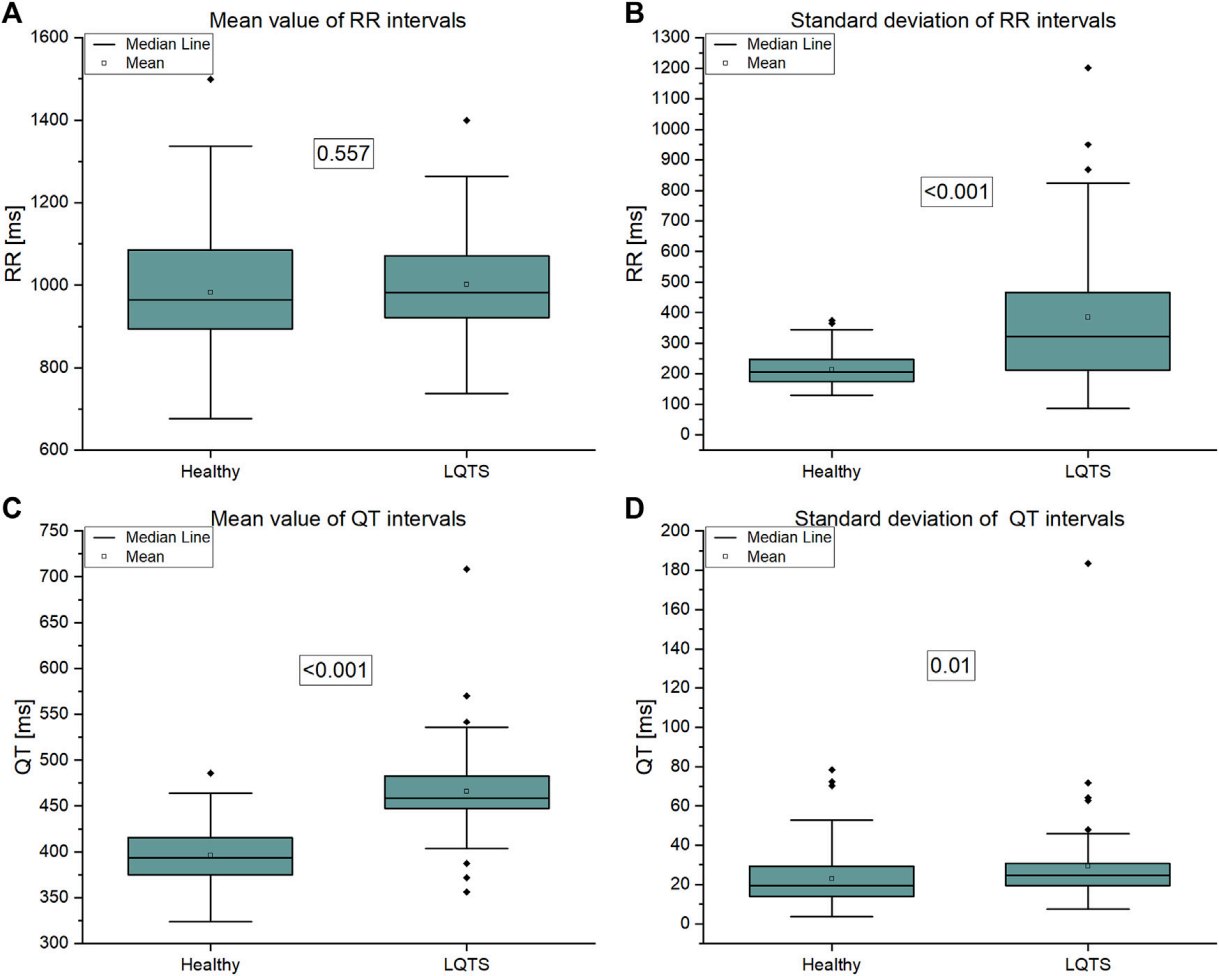
**(A)** Mean value of RR intervals time series, **(B)** Standard deviation of RR intervals time series, **(C)** Mean value of QT intervals time series, **(D)** Standard deviation of QT intervals time series.

The analysis on signal level using simple statistics shows that statistically significant differences between groups are present in QT mean (which follows from the definition of LQTS) and in both standard deviations, which are greater in LQTS group (Figure 6).

**FIGURE 7:**
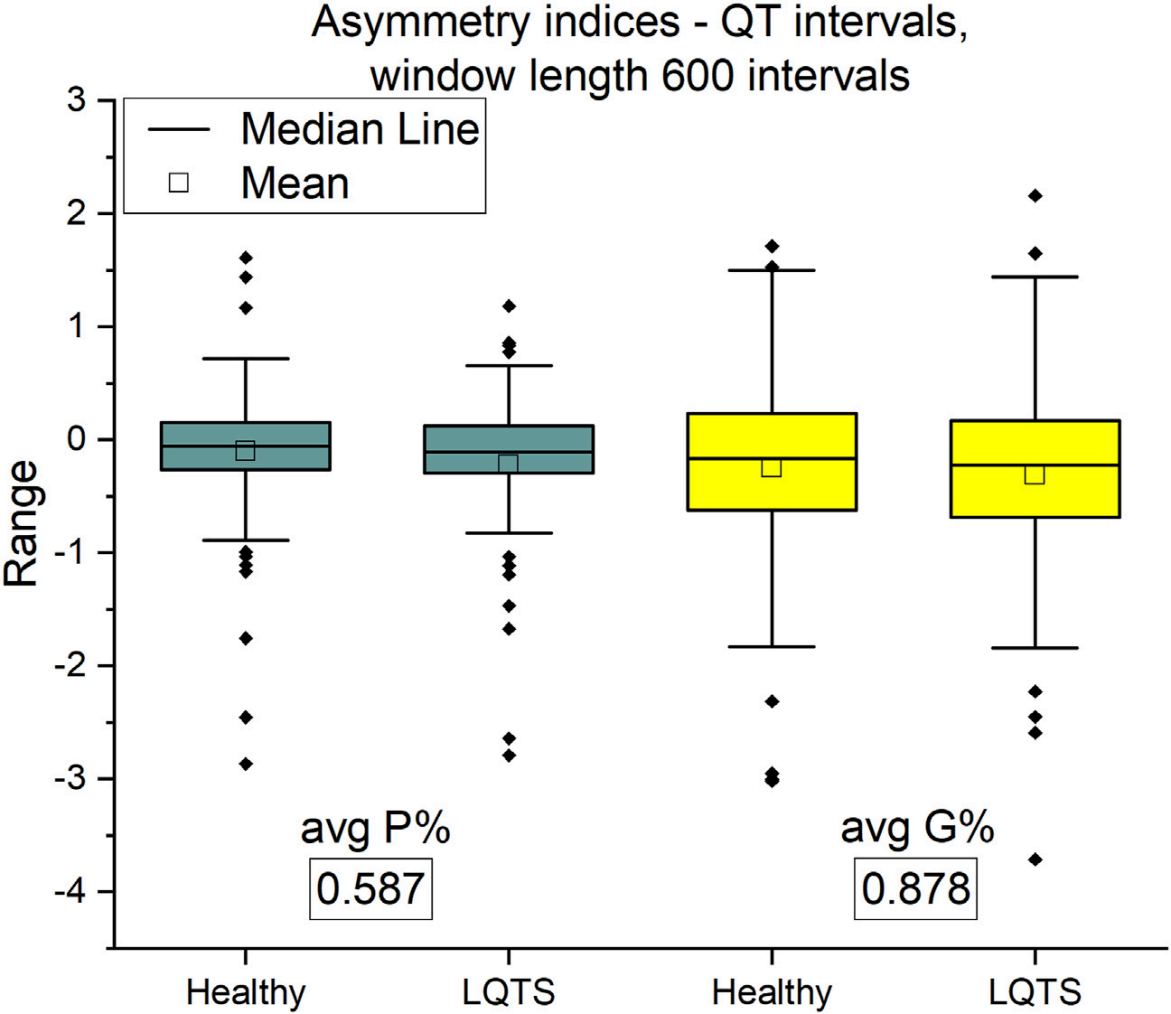
Porta *P*% and Guzik G% indices for QT intervals. Window length equals 600 intervals. *p*-values for each comparison are presented in boxes below the name of the given irreversibility parameter.

Asymmetry indices are based on differences between adjacent values of time series intervals. On the contrary, KLD estimates time irreversibility using number of points that each value of time series could reach without crossing with other points (Li et al., 2021). The asymmetry indices show no difference between groups (Figure 7). Therefore, we are interested in more complicated descriptors of dynamics of time series.

In the case of VG, we compare the average and maximum values obtained from the calculations in non-overlapping windows of the selected intervals. Because in many cases the minimum values were close to zero, they were omitted from the results.

**FIGURE 8:**
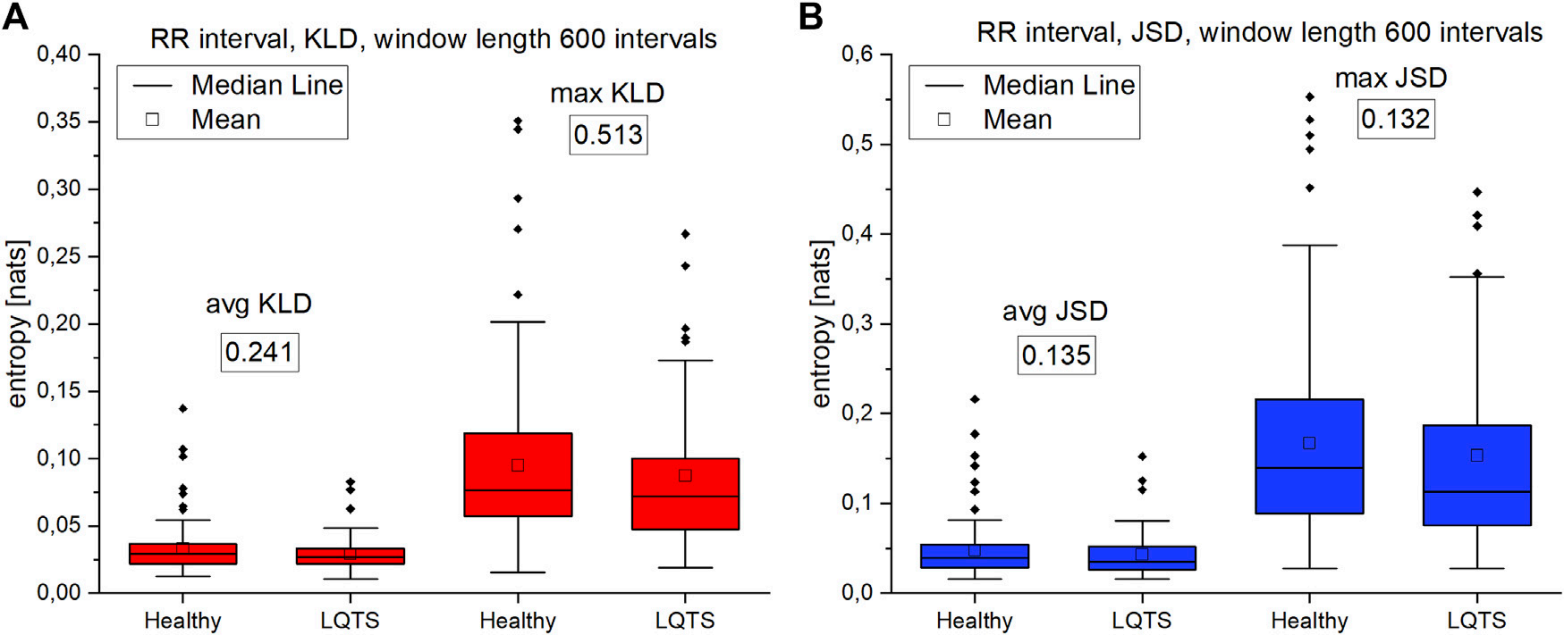
**(A)**—Kullback-Leibler divergence (KLD) for RR intervals. **(B)** Jensen-Shannon divergence (JSD) for RR intervals. Window length equals 600 intervals. *p*-values for each comparison are presented in boxes below the name of the given irreversibility parameter.

**FIGURE 9:**
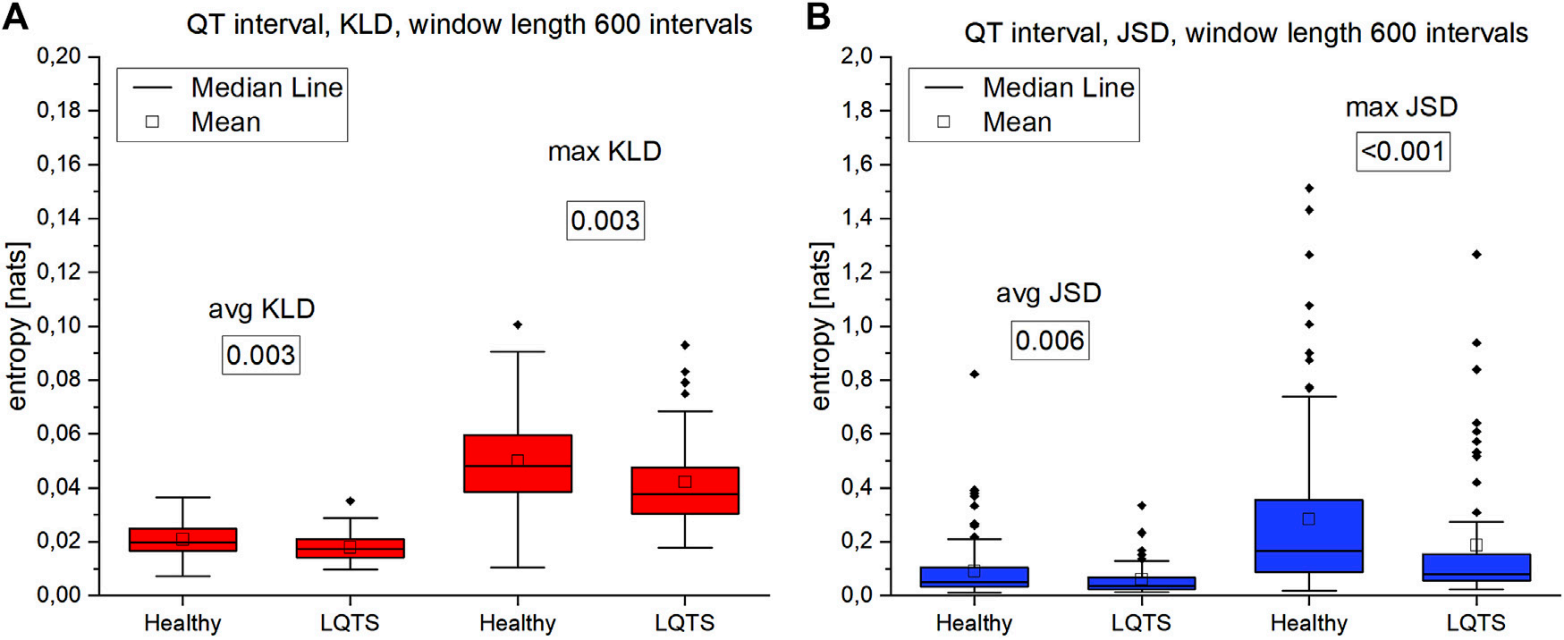
**(A)**—Kullback-Leibler divergence (KLD) for QT intervals. **(B)** Jensen-Shannon divergence (JSD) for QT intervals. Window length equals 600 intervals. *p*-values for each comparison are presented in boxes below the name of the given irreversibility parameter.

The comparison of the results for RR intervals for the healthy subjects with the patients with LQTS indicates that there are no statistically significant differences (Figures 8A,B), which would indicate no influence of the studied disease on the irreversibility of the heart rhythm. On the other hand, differences manifest themselves in the case of QT intervals (Figures 9A,B). Healthy persons are characterized by statistically significant greater irreversibility with respect to time of the QT intervals than that of the patients with LQTS.

However, there are also significant differences between the KLD and JSD for the RR and QT intervals. In the former case, they are lower. For healthy persons, the median for average KLD for the RR intervals is 0.01984 nats and median for average JSD is 0.05071 nats, while for QT it is respectively 0.01725 nats and 0.03468 nats.

**FIGURE 10:**
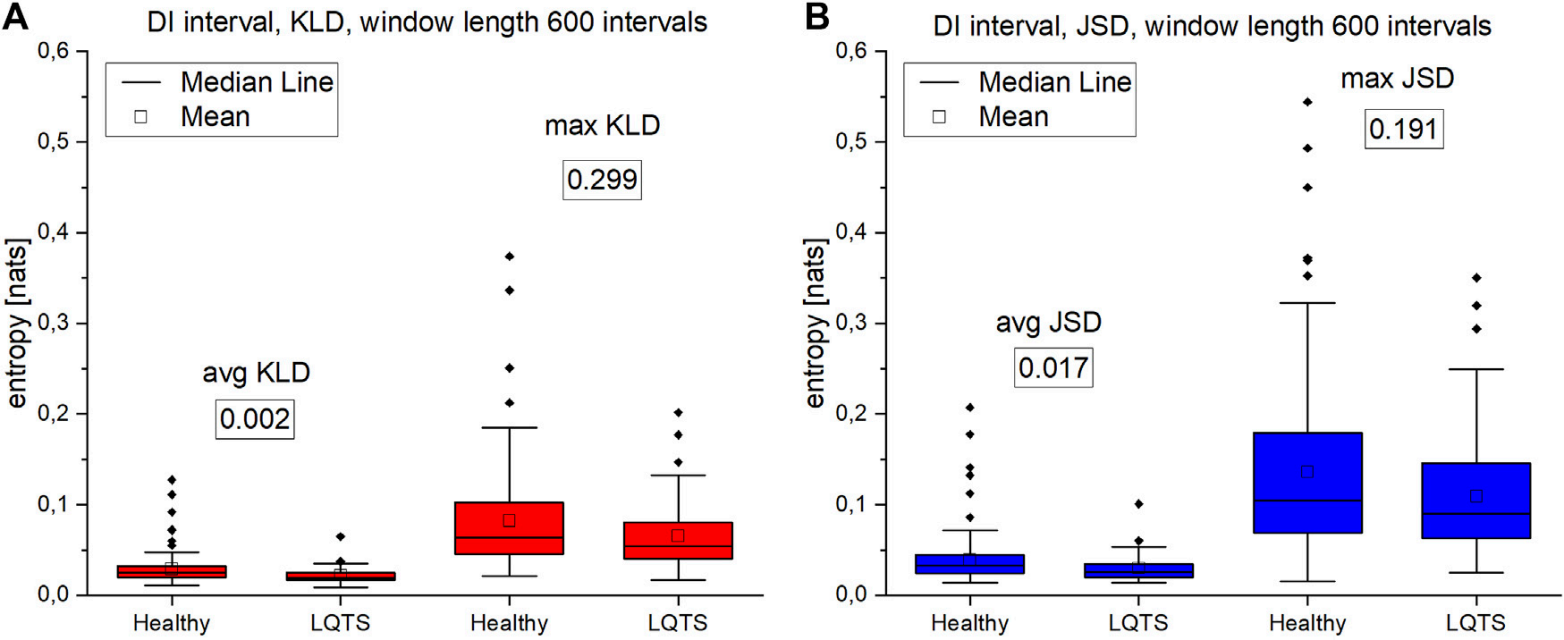
**(A)**—Kullback-Leibler divergence (KLD) values for DI intervals. **(B)** Jensen-Shannon divergence (JSD) values for DI intervals. Window length equals 600 intervals. Values presented in boxes on charts are corresponding *p*-values. *p*-values for each comparison are presented in boxes below the name of the given irreversibility parameter.

The analysis of max values of KLD and JSD using the DI intervals (Figures 10A, B) follows the results obtained for the RR time series, which is consistent with the results in (Ozimek et al., 2021). There is no difference in the irreversibility between analyzed groups. However, in the case of average KLD and average JSD here we observe higher values for the healthy indicating a larger irreversibility for the healthy.

For night recordings, statistically significant differences with the use of VG were obtained only in the case of mean and maximum values of KLD and JSD for the time series of QT intervals. However, there are no differences in the irreversibility in time between the healthy and the LQTS patients for heart rate variability. In the statistically significant cases presented above, healthy persons are characterized by a larger value of irreversibility with respect to time.

### 4.2 Assessing time irreversibility of nighttime recordings using multivariate time series

**FIGURE 11:**
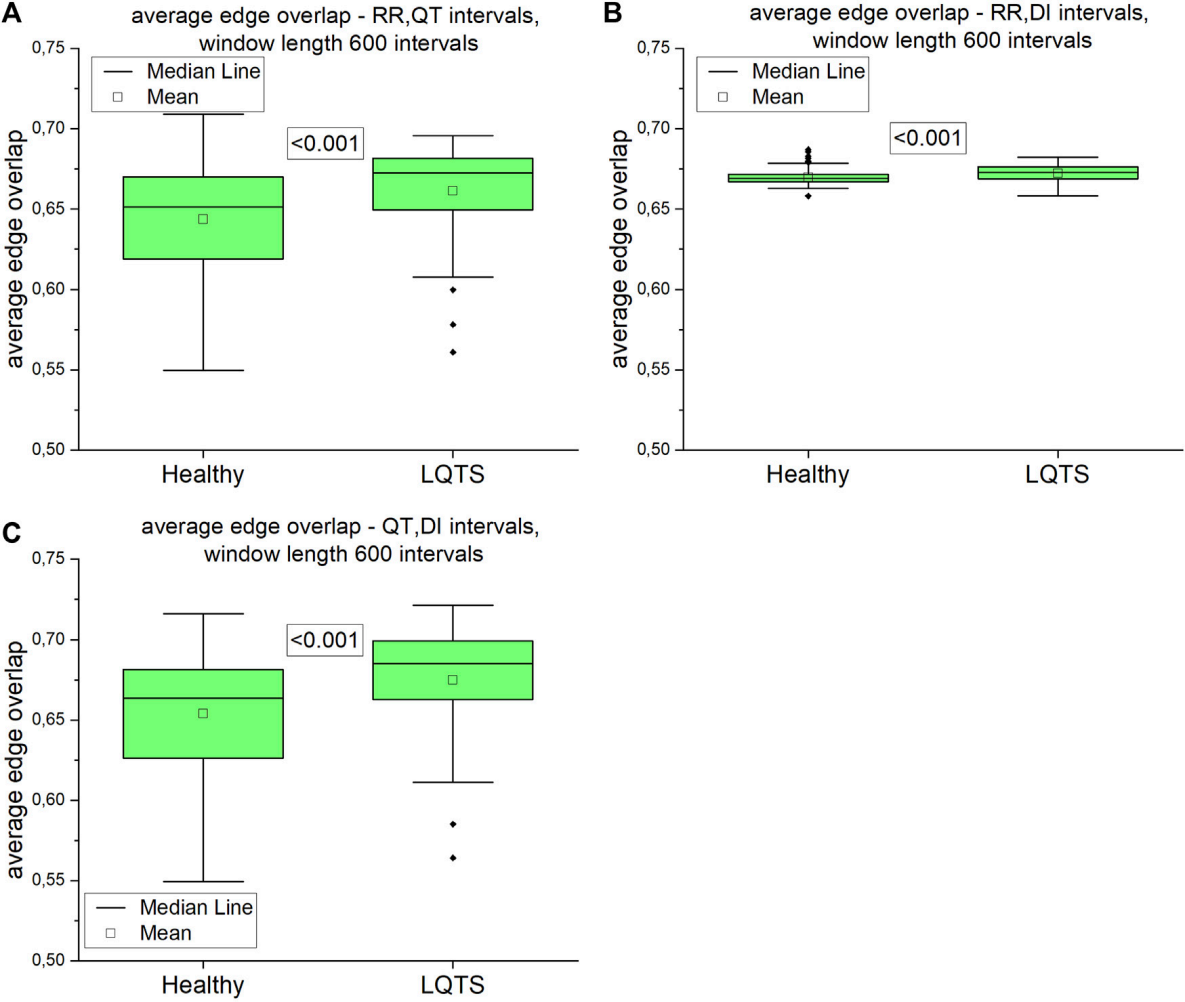
Average edge overlap calculated for pairs of time series: **(A)** RR and QT intervals time series, **(B)** RR and DI intervals time series, **(C)** QT and DI intervals time series. Using the Kolgomorov-Smirow test, we obtained the *p*-values presented in the boxes. The window length equals 600 intervals.

Our next step was to analyze multivariate time series. First, we calculated the average edge overlap between two of the three analyzed intervals. The results are presented on Figure 11.

The difference between average edge overlaps for the healthy and LQTS patients is present in all pairs of time series. The edge overlaps for LQTS patients are larger, which indicates that the graphs from these time series are more similar in all the group.

**FIGURE 12:**
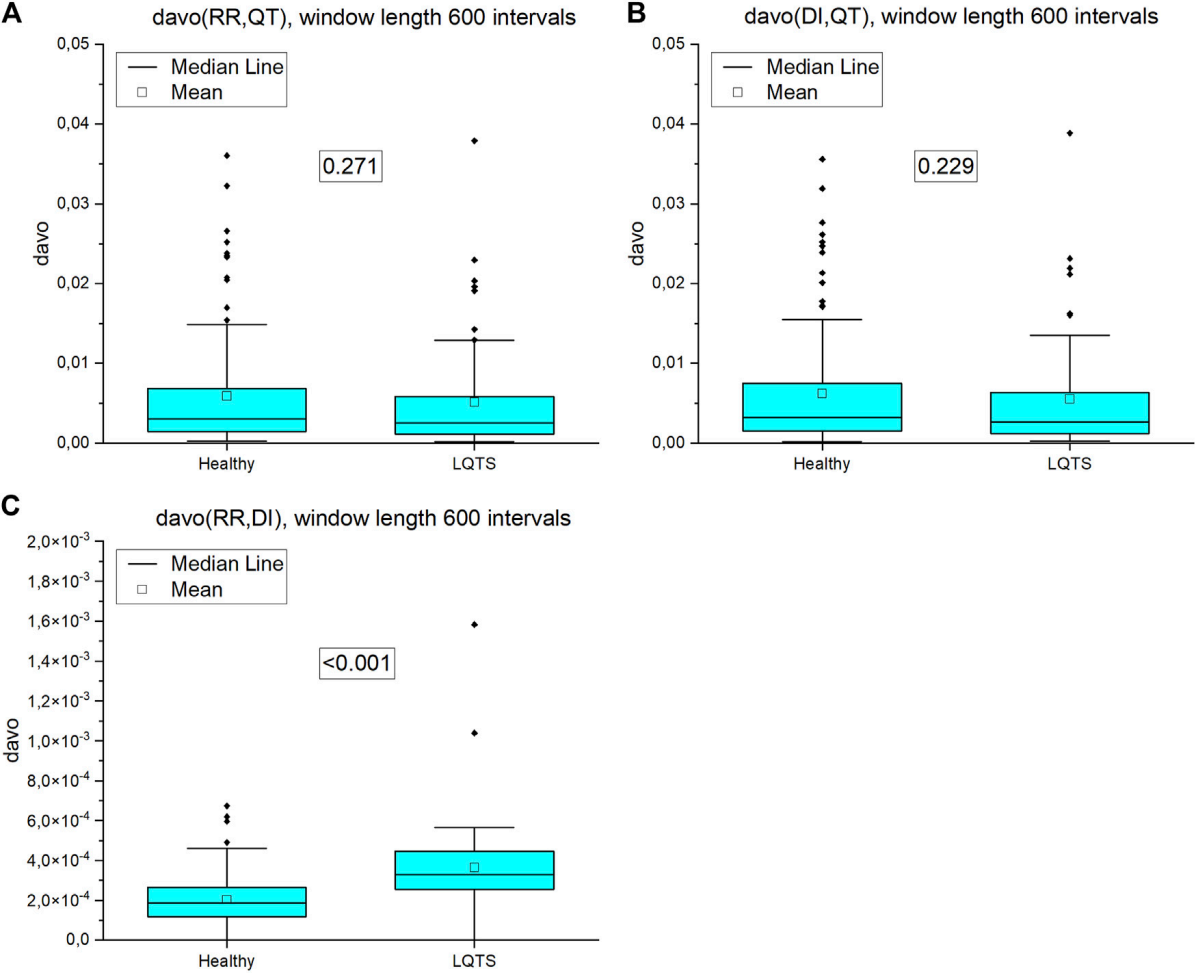
Directed average edge overlap (davo) calculated for pairs of nighttime series (window length 600 intervals): **(A)** RR and QT intervals time series, **(B)** DI and QT intervals time series, **(C)** RR and DI intervals time series. *p*-values for each comparison are presented in the boxes.

Directed average edge overlap, davo (Eq. 14) equals zero for reversible signals, the greater the value of davo , the more irreversible the signal, as the degree of layer similarity will vary depending on the direction of the passage of time. In the case of significant statistical differences, the davo obtained is, on the average, larger for LQTS patients (Figure 12), however we noticed this behavior only for the pair RR and DI intervals, where davo is much lower than for RR, QT and QT, DI intervals. The differences between groups observed in the case of average edge overlap are significantly reduced when davo is calculated, which indicates the level of irreversibility of the selected time series pairs.

**FIGURE 13:**
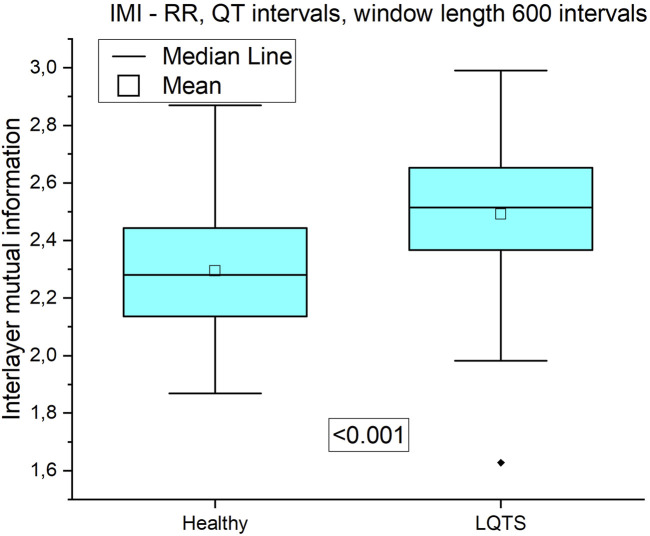
Interlayer mutual information of RR and QT time series.

For our data, only in the case of the comparison interlayer mutual information of the RR and QT intervals, we obtained statistically significant results (Figure 13). Interlayer mutual information was larger for the LQTS group.

## 5 Discussion and conclusion

It should be noted, that for the several measures presented above, we were able to obtain a statistical significance. This proves that the arrhythmogenic substrate manifests itself in irreversibility measures, which is the research hypothesis of this paper. This result encourages us to design a prospective study, in which the irreversibility measures will be correlated with the clinical findings in the follow-up period, to directly assess cardiac mortality. Irreversibility measures have proved themselves to be good candidates for such a study.

In this paper, we used signals divided into non-overlapping windows of length of 600 intervals. Such short cardiovascular series are processed to assess the short-term regulation of heart rate variability (Cohen and Taylor, 2002; Porta et al., 2009)

Summarizing the results of using VG for univariate time series, we obtained a statistically significant difference between the healthy and LQTS patients in the time irreversibility of QT intervals. The time irreversibility of QT intervals is larger for the healthy. This difference is larger for the maximum values of both KLD (Figure 9A) and JSD (Figure 9B). Moreover, using the Jensen-Shannon divergence gives a better group differentiation in this case. However, no significant difference between the groups was obtained for the heart rhythm. The choice of divergence is also important: changing from KLD to JSD results in a better differentiation of the groups (i.e., a lower *p*-value) for RR and QT intervals.

For multivariate time series, when the average edge overlap was analyzed, the connection patterns between RR and QT intervals were more like each other for the LQTS patients than for the healthy. However, when we introduce time irreversibility, namely in the form of directed average edge overlap, the results change. In this case, we did not obtain a statistically significant difference for pairs of the RR and QT and as well as the DI and QT intervals. Interlayer mutual information shows that the degree distributions between HVG obtained for the RR and QT intervals are more correlated for the LQTS patients. The presence of nonstationarities can affect the results for interlayer mutual information (Hoyer et al., 2005). Before analysis, trend-like nonstationarities were removed from the signals using EMD (Hoyer et al., 2005).

Jiang et al. (2013) found that the degree distribution of VG of RR intervals changes during meditation, which corresponds to an adjustment of the autonomous neural system. Here, we compare the difference in the degree distribution according to the direction and opposite to the direction of the time arrow. This difference used to calculate KLD does not change in LQTS subjects for the heart rate, whereas a difference between the groups occurs for QT intervals. On the contrary, multivariate methods show that the similarity of these dynamics in pairs of values is greater for individuals with LQTS, while after considering the opposite direction in time, i.e., estimating the irreversibility of such similarity, it turns out that the only difference is for the pair RR, DI where the direction of similarity is also preserved, i.e., it is greater for individuals with LQTS.

A direct comparison of the results obtained for different time series intervals can be difficult, because two systems, which have similar 1/f scaling may have different level of complexity (Ivanov et al., 2009). Ivanov et al. showed that comparing healthy people with a group with cardiopulmonary instability expresses different power-law scaling behavior (Ivanov et al., 1996). However, Mathias et al. performed a population study (Mathias et al., 2013), where 1,206 patients with LQTS were studied. The results shows that the estimated higher QTc (QT corrected for heart rate) intervals variation can be associated with a higher risk of cardiac events. This phenomenon depends on which gene was mutated and it is greatest for persons with LQTS1. In the case of QT, we observe lower values of KLD, i.e., a smaller level of irreversibility for patients with LQTS.

The measures presented in this paper do not allow a risk stratification in the LQTS group, due to insufficient patient information. However, knowing which of these parameters has the highest statistical power concerning distinguishing the groups, it is possible to define them as candidates for the identification of a clinical parameter to support the work of physicians, especially in the evaluation of SCD.

## Data Availability

Publicly available datasets were analyzed in this study. This data can be found here: The data analyzed in this study is subject to the following licenses/restrictions: Both data sets belong to the THEW Project (http://thew-project.org/databases.htm) available upon registration. Requests to access these datasets should be directed to http://thew-project.org/databases.htm.
